# Comparative genomics of five *Valsa* species gives insights on their pathogenicity evolution

**DOI:** 10.1093/g3journal/jkac312

**Published:** 2022-12-01

**Authors:** Guangchao Sun, Shichang Xie, Lin Tang, Chao Zhao, Mian Zhang, Lili Huang

**Affiliations:** State Key Laboratory of Crop Stress Biology for Arid Areas, College of Plant Protection, Northwest A&F University, Yangling 712100, Shaanxi, China; Department of Agronomy and Horticulture, University of Nebraska-Lincoln, Lincoln, NE 68588, USA; State Key Laboratory of Crop Stress Biology for Arid Areas, College of Plant Protection, Northwest A&F University, Yangling 712100, Shaanxi, China; State Key Laboratory of Crop Stress Biology for Arid Areas, College of Plant Protection, Northwest A&F University, Yangling 712100, Shaanxi, China; State Key Laboratory of Crop Stress Biology for Arid Areas, College of Plant Protection, Northwest A&F University, Yangling 712100, Shaanxi, China; State Key Laboratory of Crop Stress Biology for Arid Areas, College of Plant Protection, Northwest A&F University, Yangling 712100, Shaanxi, China; State Key Laboratory of Crop Stress Biology for Arid Areas, College of Plant Protection, Northwest A&F University, Yangling 712100, Shaanxi, China

**Keywords:** *Valsa* species, apple canker disease, comparative genomics, secondary metabolism

## Abstract

*Valsa* is a genus of ascomycetes within the Valsaceae family. This family includes many wood destructive pathogens such as the well known *Valsa mali* and *Valsa pyri* which cause canker diseases in fruit trees and threaten the global fruit production. Lack of genomic information of this family is impeding our understandings about their evolution and genetic basis of their pathogenicity divergence. Here, we report genome assemblies of *Valsa malicola*, *Valsa persoonii*, and *Valsa sordida* which represent close relatives of *Valsa mali* and *Valsa pyri* with different host preferences. Comparative genomics analysis revealed that segmental rearrangements, inversions, and translocations frequently occurred among *Valsa* spp. genomes. Gene families that exhibited gene copy expansions tended to be associated with secondary metabolism, transmembrane transport, and pyrophosphatase activities. Orthologous genes in regions lost synteny exhibited significantly higher rate of synonymous substitution (KS) than those in regions retained synteny. Moreover, among these genes, membrane transporter families associated with antidrug (MFS, DHA) activities and nutrient transportation (SP and APCs) activities were significantly over-represented. Lineage specific synonymous substitution (KS) and nonsynonymous substitution (KA) analysis based on the phylogeny constructed from 11 fungal species identified a set of genes with selection signatures in *Valsa* clade and these genes were significantly enriched in functions associated with fatty acid beta-oxidation, DNA helicase activity, and ATPase activity. Furthermore, unique genes that possessed or retained by each of the five *Valsa* species are more likely part of the secondary metabolic (SM) gene clusters. SM gene clusters conserved across five *Valsa* species showed various degrees of diversification in both identity and completeness. All 11 syntenically conserved SM clusters showed differential expression during the infection of apple branch with *Valsa mali* suggesting involvements of secondary metabolism in the pathogenicity of *Valsa* species.

## Introduction

*Valsa* spp., a fungal genus of Ascomycota phylum, includes several destructive woody canker pathogens that infect apple, pear, poplar, cherry peach, and many other economically or ecologically important rosids (Jérôme Collemare 2016). *Valsa mali*, a causal agent for apple canker disease, has become notorious for causing huge yield losses of seasonal apple production in eastern Asia (Li *et al.* 2015; Wang *et al.* 2015). In areas with severe incidence, apple gardens can be deteriorated and it takes years to recover. Recent efforts taken to understand the molecular mechanisms underlying the pathogenecity have paved a way toward effective strategies for disease control (Ke *et al.* 2013; Peng *et al.* 2016). However, likely due to a highly deversed genetic pool and heterotha, frequent emergence of new pathovars imposes big challenges to current strategies for disease control. Comparative genomics studies on *Valsa mali* and *Valsa pyri* suggested genomic adaptation substantially contributed to their virulence (Wang, Zang, *et al.* 2014; Yin *et al.* 2015). A significant change in the content of genes involved in cell wall degradation, host immunity suppression, and secondary metabolite biogenesis and repeat sequences were observed since their divergence from the common ancestor 4 million years ago (Yin *et al.* 2015).

**Fig. 1.**
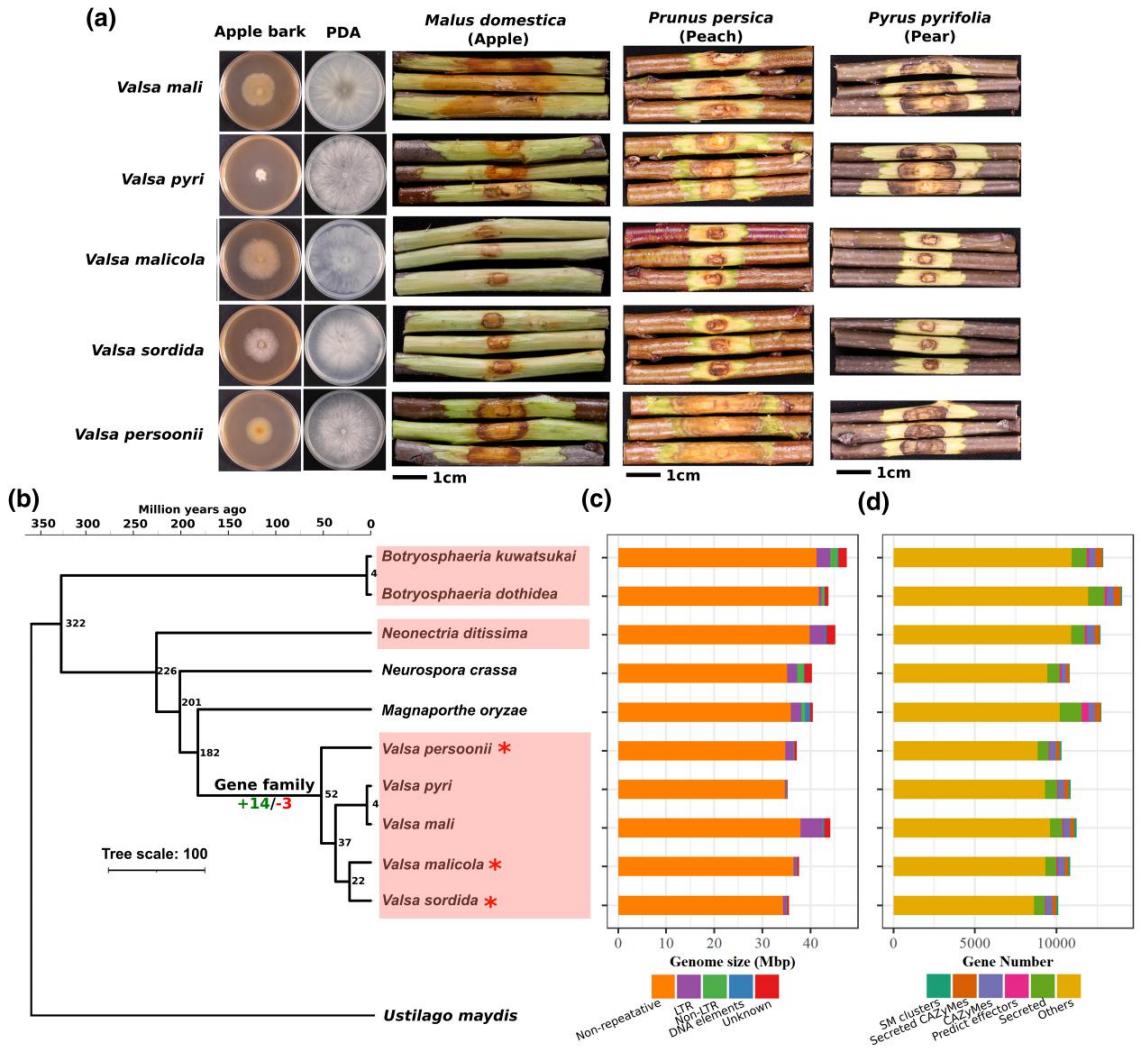
Genome and gene family evolution of five *Valsa* species genomes. a) Axenic growth and woody tree branch infection by five *Valsa* species. Scale bars are 1 cm, images were taken 7 days after inoculation. b) Genus level phylogeny and split time estimation of five *Valsa* species and related fungal species. Node labels are the estimated split time (million years ago). Species names with asterisk are the species sequenced in this study. c) Genome size and the proportion of different repeat sequence families including DNA elements, Non-LTR, LTR, and Nonrepetitive elements. d) Number of genes annotated as secondary metabolic clusters, secreted CAZyMes, unsecreted CAZyMes, predicted effectors, secreted proteins, and other functions.

*Valsa* species exhibited obvious host preference (Fig. 1a). As mentioned hereinbefore, *V. pyri* and *V. mali* diverged from the common ancestor at 4 million years ago and now exhibit obvious preferential infection on apple and pears, respectively (Fig. 1a). Host preference specification is a consequence of population competition which drives genomic selection (Abdullah *et al.* 2017). Other *Valsa* species such as *V. malicola*, primarily found on apple trees with low pathogenicity, has been reported to be more pathogenic on poplar in Europe (Wang *et al.* 2011, 2020); *V. persoonii* infects poplar (Populus spp.) primarily but was also isolated from apple trees suffering canker diseases (Wang *et al.* 2007); *V. sordida* mainly infects peach or cherry (*Prunus* spp.) (Kobayashi 1970; Adams *et al.* 2006; Wang *et al.* 2020) but was also reported to be a causal agent of sinusitis in immune-compromised human suffering acute myeloid leukemia (Kalkanci *et al.* 2006). Due to a wide range of woody hosts and their mode of infection, Valsa canker has become a major threat to global fruit production.

In this study, we report genome assemblies and annotations for three *Valsa* species, *Valsa malicola*, *Valsa persoonii*, and *Valsa sordida*. Along with two previously sequenced *Valsa* species *V. mali* and *V. pyri*, six non-Valsa fungal relatives including *Neurospora crassa*, *Magnaporthe oryzae*, *Neonectria ditissima*, *Botryosphaeria dothidea*, *Botryosphaeria kuwatsukai*, and *Ustilago maydis*, we constructed a maximum likelihood phylogeny and estimated lineage divergent time. Comparative genomics analysis based on the phylogeny constructed revealed that 14 gene families associated with secondary metabolic process, transmembrane transporter activities were specifically expanded in *Valsa* species. Orthologous genes in regions lost synteny among *Valsa* species showed significantly higher level of sequence differentiation (synonymous substitution rate, KS) and tend to be enriched in functions related to membrane transporter activities, secondary metabolic process, fungal-type cell wall biogenesis, and antioxidation. The highly conserved secondary metabolic gene clusters across five *Valsa* species showed significant transcriptional changed during the infection of apple tree bark by *Valsa mali* suggesting their roles in pathogenicity.

## Materials and methods

### Phylogeny based on whole genome single-copy orthologues

Single-copy proteomes were aligned by mafft using –auto mode. The alignments were then trimmed by Gblocks using the parameters “-t=p -b1=5 -b2=6 -b3=8 -b4=10 -a=y” to obtain the conserved blocks. The conserved blocks identified by Gblocks were then concatenated. Phylogeny with branch length was constructed by IQ-TREE (Nguyen *et al.* 2015) using the parameters “-nt AUTO -m MFP -bb 1000.” Strains of five *Valsa* species were used in this study. *Valsa mali* isolate 03-8 and *Valsa malicola* isolate 03-1 were isolated from apple trees; *Valsa pyri* was isolated from pear trees; SXYL134, a *Valsa persoonii* (= leucostoma) isolate SXYLt was isolated from a peach tree, and a *Valsa sordida* isolate YSFL was isolated from a poplar tree. All of the above strains were provided by the State Key Laboratory of Crop Stress Biology in Arid Regions of Northwest A & F University as PDA medium cultures. All of the strains were revived for a consecutive of two generations in a dark room at 25° for 48 h to fully recover the pathogenicity.

### Branch inoculation assay

Peach, pear, and apple tree branches with similar thickness were cut into sections of length 10 cm and rinsed by regular tap water. They were then soaked in 6% sodium hypochlorite solution for 15 min followed by three times of rinsing by sterilized tap water to sterilized branch surfaces. After air drying, the ends of the branches were sealed by paraffin wax. Each of the branches were burnt wounded by a soldering gun with a 5 mm punch. Revived colonies of each of the five *Valsa* species were inoculated to the wounds. Sterile PDA media punches were inoculated to the wounds as negative controls. The inoculated branches were then placed in containers with high internal moisture at 25° for 7 days. Lesion lengths were then measured for each of the inoculated branches. Five wounded branches of three tree species were inoculated with each of the five *Valsa* species. Experiments were conducted in triplicates.

### Genome sequencing, assembly, and gene prediction

An Illumina HiSeq sequencing platform was used to sequence the whole genome of three strains. Paired-end reads generated achieved sequencing depths of 147.3x, 154.1x and 195.6x for *Valsa malicola*, *Valsa persoonii*, and *Valsa sordida*, respectively. ABySS version 1.9.0 (Simpson *et al.* 2009) was used to perform De Novo assembly of the genome. Assembly completeness was evaluated using Benchmarking Universal Single-Copy Orthologs (BUSCO) 2.0 (Simão *et al.* 2015). The protein-coding genes were annotated using MAKER version 2.31.8 (Cantarel *et al.* 2008), and the completeness of the predicted proteome was also evaluated using BUSCO 2.0 (Simão *et al.* 2015).

### Repeat sequences and AT-rich regions

Repeat sequences of five strains were detected using a combination of REPETMODELER and REPEATMASKER (Tarailo-Graovac and Chen 2009). First, REPEATMODELER was used to perform De Novo prediction of repeat sequences. The obtained consensus.fa file was then integrated into fungal RepBase (Bao *et al.* 2015). The integrated database was then used as the input of REPEATMASKER for repeat sequences prediction in five *Valsa* genomes. Content differences of different repeat types across species were calculated using a in-house Perl script. OcculterCut version 1.1 (Testa *et al.* 2016) was used to annotate AT-rich regions of five strains.

### Genome annotation

Nonredundant (NR) database-based annotation was performed using The National Center for Biotechnology Information (NCBI) online BLAST service. InterPro database-based annotation was performed using the Perl script, iprscan5.pl (Mulder and Apweiler 2007). The two sets of annotation were then combined and used as input of Blast2GO version 4.0 to obtain GO function annotations (Conesa *et al.* 2005). The Kyoto Encyclopedia of Genes and Genomes (KEGG) (Kanehisa and Goto 2000) online service (https://www.kegg.jp/blastkoala/) was used to annotate KEGG metabolic pathways. Pfam 30.0 (Finn *et al.* 2014) database was used to annotate protein domains locally by running pfamscan.pl. Potential pathogen-related proteins were predicted using an online service (http://phi-blast.phi-base.org) provided by the Pathogen host interactions (PHI) database. SignalP version 4.1 (Petersen *et al.* 2011) (http://www.cbs.dtu.dk/services/SignalP/) was used to predict secretion signals of proteins. Transmembrane domains of proteins with secretion signals were predicted by TMHMM version 2.0 (http://www.cbs.dtu.dk/services/TMHMM-2.0/) (Krogh *et al.* 2001). If a protein predicted to possess secretion signals while no transmembrane region was found 30 amino acids after the secretion signal, the protein was then fed into EffectorP version 2.0 (Sperschneider *et al.* 2018) to test the possibility of being an effector protein. Annotations of cell wall degrading enzymes were retrieved by hmmscan using the CAZymes HMM model file provided by dbCAN (Yin *et al.* 2012), and the results were classified and counted by the hmmscan-parser.sh script. Protease genes were searched in the MEROPS database (Rawlings *et al.* 2002, 2018) using Blastp. Transporter Collection Database (TCDB) (Saier *et al.* 2006) was used to identify membrane transporters. Lipase genes were searched in the Lipase database (http://www.led.uni-stuttgart.de/) using Hmmscan (Eddy 2011). Secondary metabolic gene clusters were predicted using the antiSMASH version 4.2 (Blin *et al.* 2017). The predicted gene clusters were combined with InterPro annotations and uploaded to the Secondary Metabolites by InterProScan (SMIPS, https://sbi.hki-jena.de/smips/index.php) (Wolf *et al.* 2016) to identify cluster backbone genes.

### Gene family clustering analysis

BLASTP was performed for an all vs all alignment of the proteomes of the five species, and then OrthoMCL version 2.0.9 (Li *et al.* 2003) was used to find homologous gene pairs, where the parameters were set with the sequence identity threshold as 50% and e value as $1\times 10^{-5}$. Then use mcl to cluster OrthoMCL results, where the inflation value is set to 1.5. In order to clarify the phylogenetic relationship between the five strains, other six fungal relative species were introduced to obtain a set of 2,213 single-copy orthologoues using OrthoFinder (Emms and Kelly 2015). MAFFT version 7.402 (Katoh and Standley 2013) was used for aligning these gene families. The tool linsi in the multisequence alignment was used. After concatenating all the alignment results, Gblocks version 0.91b (Castresana 2000) was used to extract 794,987 conserved amino acid sites for phylogenetic tree construction. The phylogenetic tree was inferring by IQ-TREE (Nguyen *et al.* 2015) with parameters “-m MFP -bb 1000.” CAFE 5 (De Bie *et al.* 2006) was used to identify gene families undergoing expansion or contraction in *Valsa* clade.

### Synteny analysis

Synteny Mapping and Analysis Program (Symap) version 4.2 (Soderlund *et al.* 2011) was used to conduct pairwise genomic collinearity analysis. Contigs shorter than 10 kbp were filtered out, and gene pairs within collinear blocks were identified using MCScanX (Wang *et al.* 2012). Structural variations such as trans-chromosomal recombination, segmental inversion, or insertion were identified using *Valsa mali* as a reference because it is the only species with pseudo-molecule level genome assembly and annotation. Trans-chromosomal recombination event was defined as genomic regions from multiple different chromosomes of *Valsa mali* corresponding to a single contig of other *Valsa* genomes such as transposition of syntenic blocks and inversion of individual genes in the syntenic block. Segmental inversion was defined as a single chromosome of *Valsa mali* corresponding to a single contig of other *Valsa* species such as transposition and inversion of syntenic blocks or insertion and deletion of sequences between adjacent syntenic blocks.

### Branch-site positive selection analysis

ParaAT.pl (Zhang *et al.* 2012) was used to transform the amino acid multiple sequence alignment results obtained from the MAFFT alignment into codon alignment. Single-copy orthologous genome families among seven fungi (five *Valsa* species, *Magnaporthe oryae* and *Sclerotinia sclerotiorum*) were selected for analysis using the codeML tool in PAML (Yang 2007). CodeML’s branch-site model (model = 2, Nsites = 2) uses two hypotheses to analyze each branch separately. Parameters for null hypothesis (H0) were set to fix_kappa = 0, fix_omega = 1. Parameters for alternative hypothesis (H1) were set to fix_kappa = 0 and fix_omega = 0. The two hypotheses obtain the likelihood values LnL0 and LnL1, respectively. P values of 2 × (LnL1-LnL0) were calculated by chi-square test then correct for false discovery rate using the FDR (Benjamini–Hochberg Procedure) method. Genes with corrected P values lower than 0.05 were considered as under positive selection. Gene sites under positive selection were detected by Bayes Empirical Bayes (BEB) in the alternative hypothesis result.

### Transcriptional regulation of secondary metabolic gene clusters across five *Valsa* spp.

To investigate transcriptomic changes of the secondary metabolic gene clusters during the infection of apple branch by *Valsa mali*, we isolated RNA samples from the invasive hypha of *V. mali* from the colonized apple tree branches and performed RNA sequencing using an Illumina sequenser (Illumina, San Diego, CA, USA). Ten secondary metabolic gene clusters in syntenic blocks across at least three species with average identity higher than 50% were identified. TBtools (Chen *et al.* 2020) was used to fetch the coding sequences (cds) of backbone genes of these 10 secondary metabolic gene clusters. The CDS fetched by TBtools were then translated into amino acid sequences and used as queries to search for homologes in *V. malicola*, *V. persoonii*, *V. pyri*, and *V. sordida* locally constructed protein database by blastp (Johnson *et al.* 2008). The hits with E-value lower than $1\times 10^{-10}$ and identity higher than 50% were considered as orthologues. FPKM was calculated using cufflinks v2.2.1 (Trapnell *et al.* 2012). Fold change and adjusted P values comparing to control condition were calculated using DESeq2 (Love *et al.* 2014).

## Results

### Genome assembly and annotation of five *Valsa* species

Paired-end sequencing was performed with genome DNA samples isolated from *V. malicola*, *V. sordida*, and *V. persooni* by an Illumina Hiseq platform. Sequencing depth for *V.malicola*, *V.sordida*, and *V.persooni* is 147.3x, 195.6x, and 154.1x, respectively, genomic sequences were assembled by ABySS (Simpson *et al.* 2009) into 287, 260, and 353 scaffolds which represent 98.9%, 98.6%, and 98.9% of the whole genomes as evaluated by BUSCO (Simão *et al.* 2015), respectively. As predicted by MAKER, Proteomes of *V.malicola* possessed the most protein encoding genes (10,848), followed by *V. persooni* (10,296) and *V. sordida* (10,099) (Cantarel *et al.* 2008). Proteome completeness of *V.malicola*, *V. persooni*, and *V. sordida* reached 97.9%, 98.9%, and 98.6% as evaluated by BUSCO (Simão *et al.* 2015). However, these high completeness scores should be interpreted with cautions since BUSCO is likely biased toward conserved genomic regions that are relatively easy to assembly. Including the previous sequenced *Valsa mali* and *Valsa pyri*, gene number comparison of functional groups of annotated genes across five species showed little to none noticeable variations except for pathogenicity related genes (Fig. 1d, Supplementary Table 1). Despite the moderate variations in gene copy numbers, the five *Valsa* genomes showed significant size difference by ranging from 35.73 to 44.52 Mbp. This variation is largely attributable to repeat sequence contents which varied from 2.83% in *V. pyri* genome and 14.18% in *V. mali* genome (Fig. 1c). The higher content of repeat sequences in *V. mali* is likely due to frequent repeat sequence insertion into gene bodies and secondary metabolic clusters (Supplementary Table 3). It should be noted that this observation might reflect low completeness of genomic regions with high repetitive sequences in the Illumina assembles. The classification of repeat sequences in five *Valsa* species genomes based on RepeatMasker (RepBase version 20170127) and RepeatModeler (de novo prediction) suggested long terminal repeat (LTR) transposon elements (TEs) is the major content (Supplementary Table 2).

Next, we used 1,250 single-copy syntenic genes identified across 11 fungal relatives including five *Valsa* species, three additional fungal species that cause woody tree canker disease including *Botryosphaeria kuwatsukai*, *Botryosphaeria dothidea*, and *Neonectria ditissima*, two well known model filamentous fungi *Neurospora crassa* and *Magnaporthe oryzae*, and lastly a basidiomycete *Ustilago maydis* as an outgroup to construct a maximum likelihood phylogeny and estimated lineage divergent time using the divergent time between *V. mali* and *V. pyri* (4 million years ago) as a reference (Fig. 1b). In consistent with previous studies (Wang, Zang, *et al.* 2014; Yin *et al.* 2015), *V. mali* and *V. pyri* were placed together in a clade (Fig. 1b). *V.sordida* and *V.malicola* were place together in a sister clade estimated to have diverged from the *V. mali* and *V. pyri* clade at about 19 million years ago. *V.sordida* and *V.malicola* were estimated to have diverged at 9.8 million years ago. *V. persooni* was placed in a single branch as an outgroup to the other four *Valsa* species and estimated to have diverged from the other two *Valsa* clades at 28 million years ago (Fig. 1b). Interestingly, the other three canker fungi *B. kuwatsukai*, *B. dothidea*, and *N. ditissima* were placed outside of the *N. crassa* and *M. oryzae* clades which suggested a history of host switch of the common ancestor from woody plants to Poaceae (Fig. 1b).

### Evolution of gene families in *Valsa* species

**Fig. 2.**
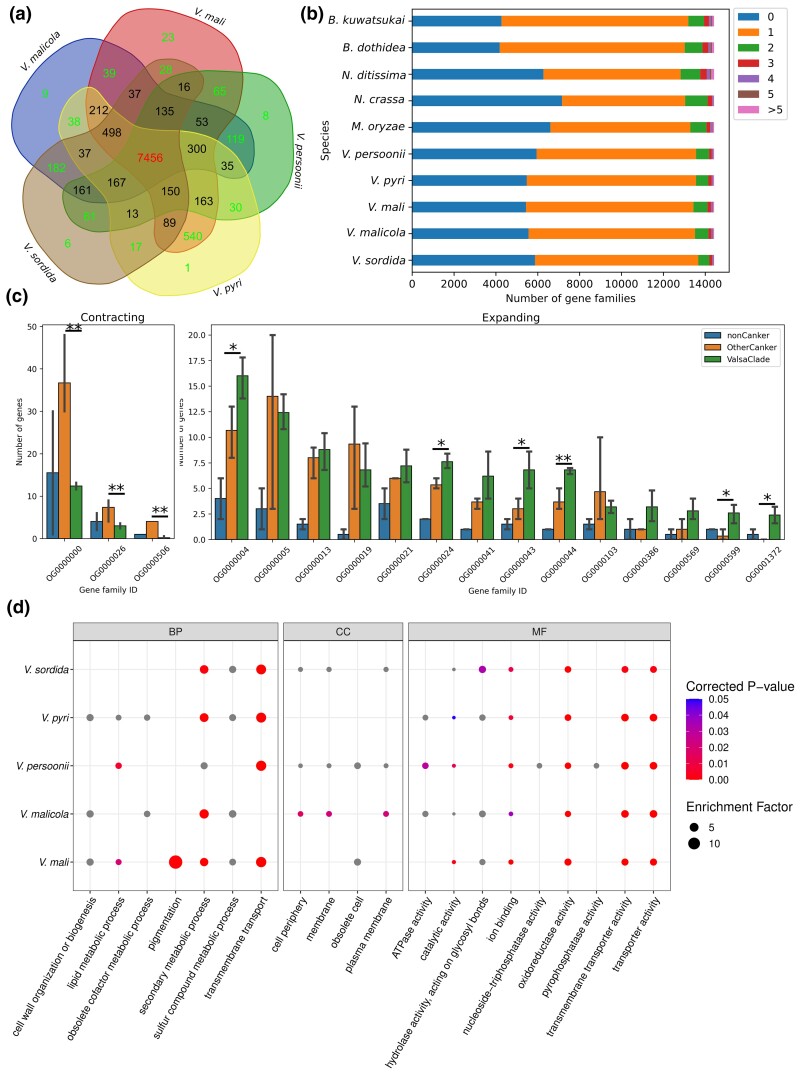
Evolution of gene families in *Valsa* species. a) Conservation and specification of gene families across five *Valsa* genomes. b) Gene families with different copy numbers of genes in the species included in the phylogeny. c) Bars indicating number of genes in the Valsa clade contracted and expanded gene families across three groups of species included in the phylogeny as shown in Fig. 1b. d) Gene ontology terms over-represented in the significantly expanded gene families in *Valsa* clade.

Annotated protein sequences for five *Valsa* species were grouped into 10,688 gene families among which 7,456 were present in five species, with the remainder being present in 1–4 species (Fig. 2a,b). Of the gene families present in all five *Valsa* species, 94% (6,980) were single copy in that species suggesting gene duplication events predominantly occurred at local rather than whole genome level. Using the phylogeny and estimated split time, CAFE (De Bie *et al.* 2006) identified three gene families undergoing contraction and 14 gene families undergoing expansion in *Valsa* spp. (Fig. 1b). Genes of the three contracted gene families are enriched in sulfur compound metabolism and secondary metabolism. Interestingly, ancestral lineages of canker fungi (*B. kuwatsukai*, *B. dothidea*, and *N. ditissima*) possessed almost two folds more gene copies of these families than other species groups (noncanker and Valsa clades) included in the phylogeny (Fig. 2c).

Among the 14 expanding gene families, six of them have significantly more gene copies than their ancestral canker relatives (Student’s t-test) (Fig. 2c). Additionally, these 14 gene families tend to be functionally related to transmembrane transport, secondary metabolic process, and pyrophosphatase activity (Fig. 2d).

To explore lineage specific diversification of genes during evolution, we identified 2,334 single-copy orthologues among *Valsa* spp., *Magnaporthe oryzae*, *Neurospora crassa* and *Ustilago maydis* using orthomcl (Li *et al.* 2003) and detected selection signatures in the *Valsa* clade using the branch-site model implemented in PAML (Yang 2007) (Supplementary Fig. 1a) (see Methods). The results showed that genes related to fatty acid beta-oxidation, DNA helicase activity and ATPase activity were over-represented in those genes with significant signals for positive selection (Supplementary Fig. 1b).

### Genomic diversification and lineage specific selection of genes in *Valsa* spp.

**Fig. 3.**
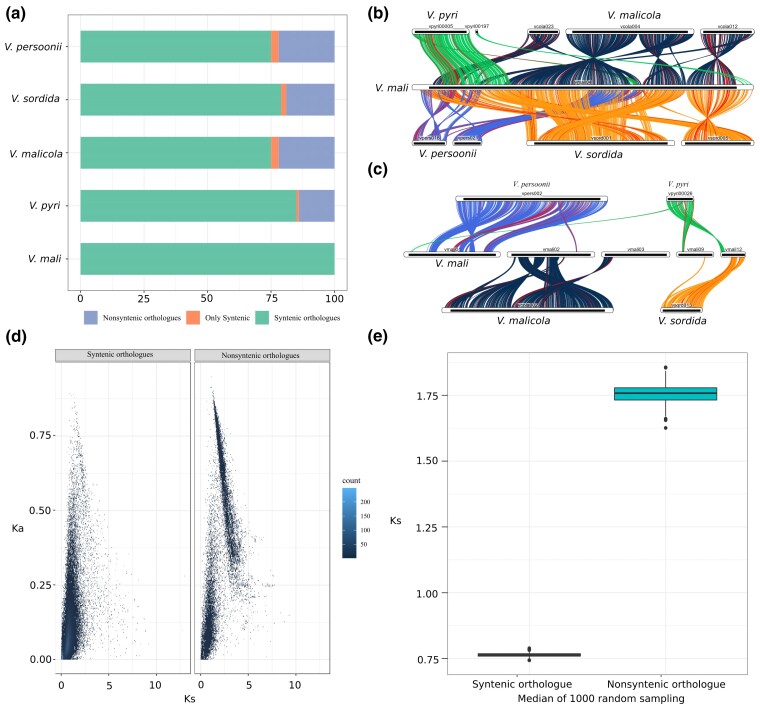
Genomic conservation and diversification across five *Valsa* species. a) Proportion of orthologous genes in syntenic regions (Syntenic orthologues) and orthologues in regions lost synteny (Nonsyntenic orthologues) with *V. mali*. Only syntenic indicates genes in syntenic regions but no orthologous pairs were found in other species. b) Examples of inter-chromosomal rearrangement events identified among four species using *V. mali* as a reference. c) Examples of intra-chromosomal rearrangement events identified among four species using *V. mali* as a reference. d) Comparison between nonsynonymous substitution (Ks) and synonymous substitution (Ka) of 3,000 genes randomly selected from syntenic orthologues (left) and nonsyntenic orthologues (right), respectively. e) Comparison of distribution of median KS of 3,000 randomly selected genes from syntenic orthologues and orthologues in regions lost synteny for 100 times.

Fungal genomes exhibit a mode of chromosomal evolution called mesosynteny featured by microsyntenic regions with randomized orientation resulted from small local chromosomal rearrangements (Hane *et al.* 2011). Comparing to *V. mali* genome, the genomes of other four *Valsa* species retained an overall more than 80 % synteny (Fig. 3a) while several inter- (Fig. 3b) and intra-chromosomal (Fig. 3c) rearrangements were also observed. Most of the duplications were consistently observed in comparisons between *V. mali* and the other four species respectively (Supplementary Fig. 2a–c). A large segmental duplication between chromosome 6 and 2 was only observed in the comparison between *V. persoonii* and *V. mali* genomes (Supplementary Fig. 2d).

A set of orthologous gene pairs in regions lost synteny exhibited sequence diversification at a significantly higher level than that of the same number of randomly selected orthologous genes in syntenic regions (Fig. 3d). To confirm the pattern observed in Fig. 3d is not due to drifted sampling, we therefore randomly selected 3,000 orthologous genes from syntenic regions and nonsyntenic regions for 100 times and plot the median of KS of two selected gene sets (Fig. 3e). The median of orthologous genes in regions lost synteny (0.75) is significantly lower than those in regions retained synteny (1.75) (Fig. 3e). Among these orthologues, those with synonymous substitution rate (Ks) higher than 1.5 were significantly enriched in functions (GO) associated with membrane transporter activities such as xenophobic transmembrane transport, ion transport, organic acid transport, and oligopeptide transport; protein binding activities such as modified amino acid binding and iron ion binding; plant signal response such as response to karrikin; antioxidation such as oxidoreductase activity, fungal-type cell wall biogenesis and secondary metabolite biosynthesis (Supplementary Fig. 3).

**Fig. 4.**
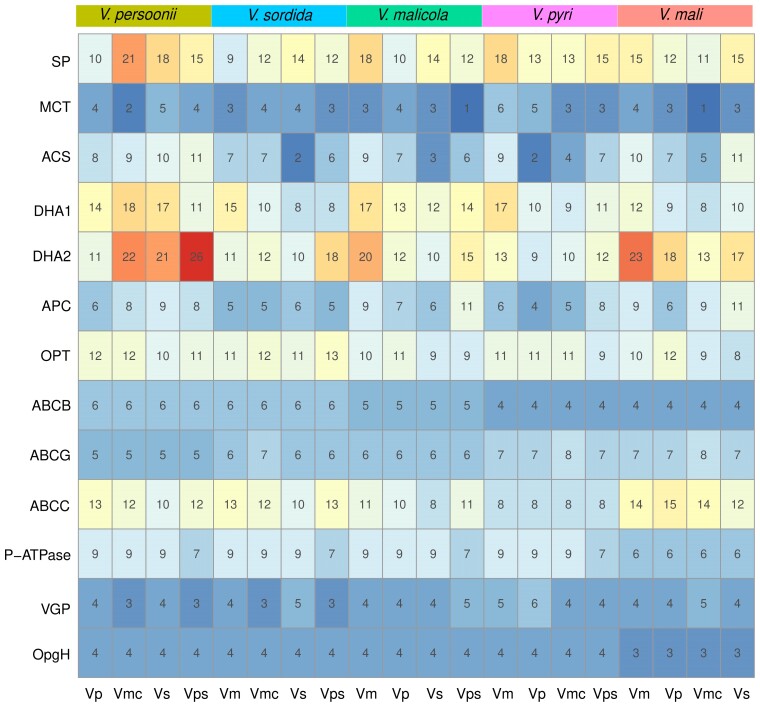
Membrane transporter family enriched in highly dynamic (KS >1.5) orthologues in regions lost synteny in *Valsa* species. Number indicates gene counts among each transporter family and color in each square indicates proportion of the counted genes in same transporter family.

Further functional classification on the set of genes associated with transmembrane transporter activities revealed that a substantial portion of which are Drug:H+ Antiporter (DHA) transporters which function as efflux pumps to confer resistance to antifungal drugs (Brôco *et al.* 1999; Teixeira *et al.* 2008) and oxidative stresses (Chen *et al.* 2017) (Fig. 4). A large number of nutrient transporter families such as sugar transporter (SP) and APCs are also included in this set of genes.

### Diversity of SM clusters and their transcriptional response during the infection of *Valsa* species

*Valsa mali* has undergone a significant SM cluster expansion compared to fungal relatives that infect grass species (Yin *et al.* 2015). Secondary metabolic clusters were identified and categorized in five *Valsa* genomes using antiSMASH using genes encoding key enzymes (NRPS, PKS and TS) as queries (Blin *et al.* 2017). Comparing to *Valsa mali*, syntenically conserved SM clusters showed different degrees of variation in both sequence similarity and cluster completeness (Supplementary Fig. 4). Terpene metabolic clusters are largely conserved while PKS clusters exhibited higher diversity (Supplementary Fig. 4). In fungal genomes, repetitive sequence and transposable element activities were contained via repeat induced C to T point mutation (RIP) to maintain genome stability leading to depletion of GC content and thus, enrichment of AT content in the genomic regions flanking repeat sequences or transposable elements (Testa *et al.* 2016; Gladyshev 2017). Therefore, AT-rich regions can be used as a proxy of repeat sequence footprint. All five *Valsa* genomes exhibited significant correlations between local repeat sequences and AT-rich regions, most repeat sequences were within 200 bp from AT-rich regions (Supplementary Fig. 5a). Two thirds of the annotated secondary metabolic gene clusters were within 5 kb away from AT rich regions in *V. mali* genome and a consistent pattern was observed in other four species (Supplementary Fig. 5b). In addition, the level of conservation relative to *V. mali* represented by cluster gene sequence similarity (Supplementary Fig. 5c) and cluster completeness (Supplementary Fig. 5d) exhibited a negative correlation with the distance to AT-rich regions.

**Fig. 5.**
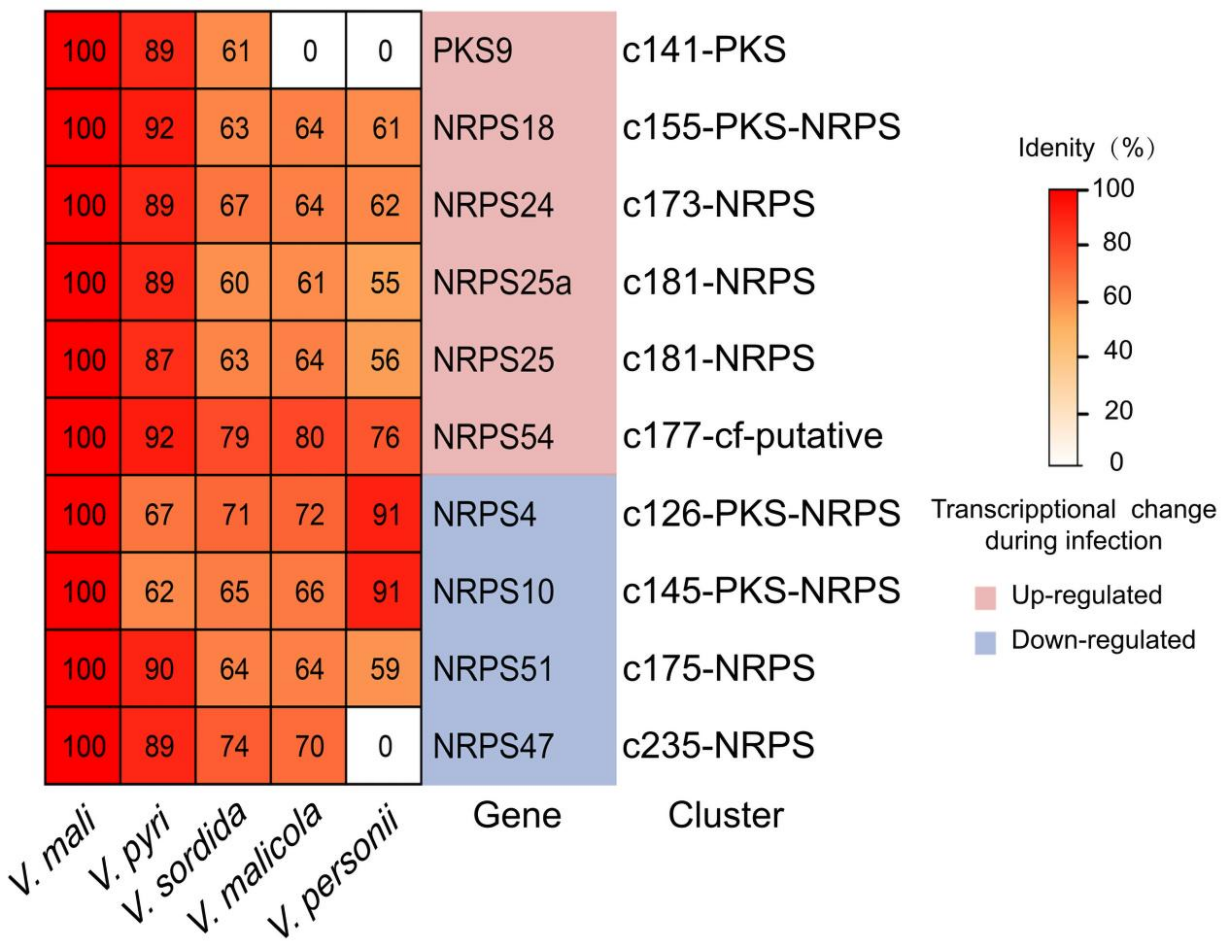
Transcriptional response of highly conserved SM clusters during the infection in *Valsa mali*. The columns in the heatmap of *V. malicola*, *V. persoonii*, *V. pyri*, and *V. sordida* indicate sequence similarity with *V.mali* secondary metabolic backbone genes, respectively. The “Gene” column indicates the backbone genes of the secondary metabolic gene clusters compared across the species. Pink and blue shades indicate up-regulation and down-regulation of the cluster during the infection of apple tree bark by *V. mali*, respectively. The cluster column indicates the secondary metabolic gene clusters that the backbone genes are located in.

*Valsa* spp. are heterotrophic pathogens that secrete various toxins synthesized by secondary metabolic clusters to kill their host cells during the early stage of infection (Wang, Li, *et al.* 2014; Doehlemann *et al.* 2017). The size and divergence of secondary metabolic clusters among the five *Valsa* genomes might be associated with the gain of ability of colonizing specific host plants. For example, reduction of pathogenicity was observed in *VmNRPS12* (Ma *et al.* 2016) and *VmNRPS14* deletion mutants (Feng *et al.* 2020). To explore more potential SM candidates that are involved in pathogenicity of *Valsa* species, we profiled transcriptoms (*unpublished*) in the invasive hypha of *V. mali* isolated from infected apple branches. Interestingly, all of the 10 SM clusters that are syntenically conserved across at least three *Valsa* species with average sequence identity over 50% showed significant differential expression during the infectious growth of *Valsa mali* (Supplementary Table 5). Backbone genes of six conserved clusters were significantly up-regulated and four of them were down-regulated (Fig. 5).

## Discussion

*Valsa* spp. is a group of destructive pathogens on woody plants, tremendous losses are caused due to its capability of infecting a variety of important woody hosts such as apple, pear, peach, and other economically important trees. The genome of *V. mali* showed that expanded pectinase families, repeat sequence insertions, toxin biosynthesis as well as effectors obtained via horizontal gene transfer are crucial contributors of its pathogenicity and drug resistance (Yin *et al.* 2015). The genome plasticity as a result of intensive genetic engagements with their host and co-exist microbe communities have risen as a promising weapon to excel local adaptation (Möller and Stukenbrock 2017). To gain insights on the evolution of pathogenicity of *Valsa* species, we performed genome sequencing of three relatives of *V. mali*, *V. persoonii*, *V. malicola*, and *V. sordida* and conducted a comprehensive comparative genomics analysis.

Comparison of the five *Valsa* genomes revealed well conserved synteny with a few segmental rearrangements and duplications (Supplementary Fig. 2; Fig. 3b). We identified some intra-chromosomal inversions featuring mesosytenies suggesting transposition of genetic elements might also be a source of genetic diversity in *Valsa* species (Ohm *et al.* 2012). However, these results need to be interpreted with cautions because among the five species, only the genome of *V. mali* was assembled and annotated to the chromosome level, the other four were only assembled to contigs which might give rise to falsely high number of inversions. In Ascomycota, microsynteny observed between their distant relatives were mostly inversion and rarely translocations (Hane *et al.* 2011), *Valsa* species might not be an exemption because we did not observe frequent translocation between long contigs that are in synteny with *V. mali* chromosomes. It should be noted that the ability to identify chromosomal translocations precisely was also limited due to a small number of long length contigs from short reads.

Gene families that are functionally associated with secondary metabolism, transmembrane transporter activity and antioxidation activities were identified with increasing gene copy numbers in *Valsa* clade based on the phylogeny constructed from 11 species (Fig. 1a). These functions are likely important for fungal pathogens to survive and thrive but these signals can also be confounded by population effects, genetic bottle necks or sampling biases. Orthologous genes in genomic regions lost synteny exhibited significantly higher than random level of sequence differentiation featured by high synonymous substitution. The orthologous genes annotated as transmembrane transporters were disproportionately higher among the ones with synonymous substitution rate higher than 1.5 (Fig. 4; Supplementary Fig. 3). Further classification of these transporters revealed enrichment of DHA transporters that are reported to be associated with fungicide resistance in fungal pathogens (Costa *et al.* 2014). Gain of genetic diversity in these genes might play roles in fungicide resistance in *Valsa* species as well. In addition, genes associated with response to plant chemical karrikin was also enriched in the orthologues in regions lost synteny. Karrikin is a group of chemical compound involved in plant seed germination and early development, it has been shown to be involved in phytohormone signaling networks (Meng *et al.* 2017). Current understandings on karrikins are primarily in plant growth, therefore, it would be interesting to further investigate whether this response to karrikin is also involved in disease defense (Meng *et al.* 2017; Antala *et al.* 2020). Species specific gene loss are usually associated with transporter and secondary metabolism that both have redundant genes in *Valsa* genomes (Supplementary Table 4). This selective but balanced gene gain and loss toward certain functions in *Valsa* species can be an important mechanism for specific environment adaptation (Casadevall 2008; Martin *et al.* 2008; Spanu *et al.* 2010).

Fungal SMs can be divided into four main chemical classes: polyketides, terpenoids, shikimic acid derived compounds, and nonribosomal peptides (Brakhage 2013). The small molecules such as fungal toxins, hormones, and other metabolites are revealed as important for fungal pathogen and even symptomless fungal endophytes for interaction with their plant hosts (Pusztahelyi *et al.* 2015). Some fungal pathogens even produce SMs to mimick the ones produced by plant host to mediate plant growth during the infection (Kazan and Lyons 2014). For example, in some cases fungal pathogens produce and secrete terpenes to manipulate host plant growth and development to support fungal growth. Conversely, it is also widely utilized by plant host as an antifungal molecule to defend infection (Collado *et al.* 2007). Polyketide synthase (PKS) and Nonribosomal peptide synthase (NRPS) have been reported to catalyze biosynthesis of secondary metabolites that facilitate infection with plant hosts by fungal pathogens (Thynne *et al.* 2019). Out of 40 SM clusters predicted in *V. mali* genome, 11 of them are sytenically conserved across at least four species and all of them showed significant change in expression during the infection of apple branch with *V. mali* (Fig. 5). These transcriptional change proved evidence of highly conserved, likely functionally necessary secondary metabolic gene clusters play critical roles in pathogenicity of *Valsa* species. Future studies focusing on the molecular mechanisms will advance our understanding of the involvement of these clusters. Last but not least, genome assemblies with better repeat sequence annotation generated from long read sequencing will provide stronger evidence of the observed association between SM diversity and AT-rich regions.

## Supplementary Material

jkac312_Supplementary_Data

## Data Availability

Genome assembly and annotation of *Valsa mali* were retrieved from NCBI with assembly ID: ASM81815v1 under BioProject: PRJNA268126; Genome assembly and annotation of *Valsa pyri* were retrieved from NCBI with Assembly ID: ASM81338v1 under BioProject: PRJNA296468; Genome assembly and annotation of *Valsa malicola* can be accessed from NCBI with Assembly ID: ASM379531v1 under BioProject: PRJNA296468; Genome assembly and annotation of *Valsa sordida* can be accessed from NCBI with Assembly ID: ASM379527v1 under BioProject: PRJNA296468; Genome assembly and annotation of *Valsa persoonii* can be accessed from NCBI with Assembly ID: ASM379529v1 under BioProject: PRJNA296468. Gene expression data for the highly conserved secondary cluster backbone genes are provided in the form of a spreadsheet as supplementary document 2. All of the data associated with the figures are included in this manuscript. Supplementary material are available at *G3* online.

## Notes

*Communicating editor: D. Baltrus*
