# Supplementary material for: Comparative genomics of five Valsa species gives insights on their pathogenicity evolution
Source: G3 (Bethesda). 2022 Dec 1;13(2):jkac312. doi: 10.1093/g3journal/jkac312 (PMC9911072; doi:10.1093/g3journal/jkac312)
Supplement: jkac312_Supplementary_Data [file jkac312_supplementary_data.zip › Supplementary_Figure_Legends_G3-2022-403750.docx]

**Figure S1 - Orthologous genes with significant selection signal in Valsa spp. branch** (A) The phylogeny used for orthologous gene pair kaks analysis. The clade labeled in red indicates the foreground clade used as targets in the codeml implemented in PAML, *Magnaporthe oryzae*, *Neurospora crassa* and *Ustiliago maydis* were used as background. (B) Gene ontology terms over-represented in the gene sets with positive selection signals in Valsa clade

**Figure S2 - Local chromosomal rearrangments of Valsa genomes** (A) Inter- and intra- chromosomal rearrangement events identified between *V. mali* and contigs larger than 50 kb of *V. pyri* assembly; (B) Inter- and intra- chromosomal rearrangement events identified between V. mali and contigs larger than 50 kb of *V. malicola* assembly; (C) Inter- and intra- chromosomal rearrangement events identified between *V. mali* and contigs larger than 50 kb of *V. sordida* assembly; (D) Inter- and intra- chromosomal rearrangement events identified between *V. mali* and contigs larger than 50 kb of *V. persoonii* assembly

**Figure S3 - Gene ontology terms over-represented in orthologous genes in regions lost synteny that under selection** (A) Gene ontology terms enriched, size indicate enrichment factor, corrected p values are color coded.

**Figure S4 - Conservation and diversification of secondary metabolic gene clusters among Valsa spp.** (A) Average protein identity between SMs and their collinearity pairs in synteny blocks. (B) The ratio of the collinearity pairs and SM genes number.

**Figure S5 - Potential role of repeat elements in SM evolution in Valsa species** (A) A significant coordination correlation between repeat elements and AT-rich regions was observed among all five *Valsa* species; (B) Number of SM detected in different distance to AT-rich regions in five *Valsa* species. (C) Relation of SM gene identities to their *V. mali* orthologues with the distance to AT-rich regions. (D) Synteny retention of SM clusters to their *V. mali* orthologues with their distance to AT-rich regions.
