# Supplementary figures and images for: Comparative genomics of five Valsa species gives insights on their pathogenicity evolution
Source: G3 (Bethesda). 2022 Dec 1;13(2):jkac312. doi: 10.1093/g3journal/jkac312 (PMC9911072; doi:10.1093/g3journal/jkac312)

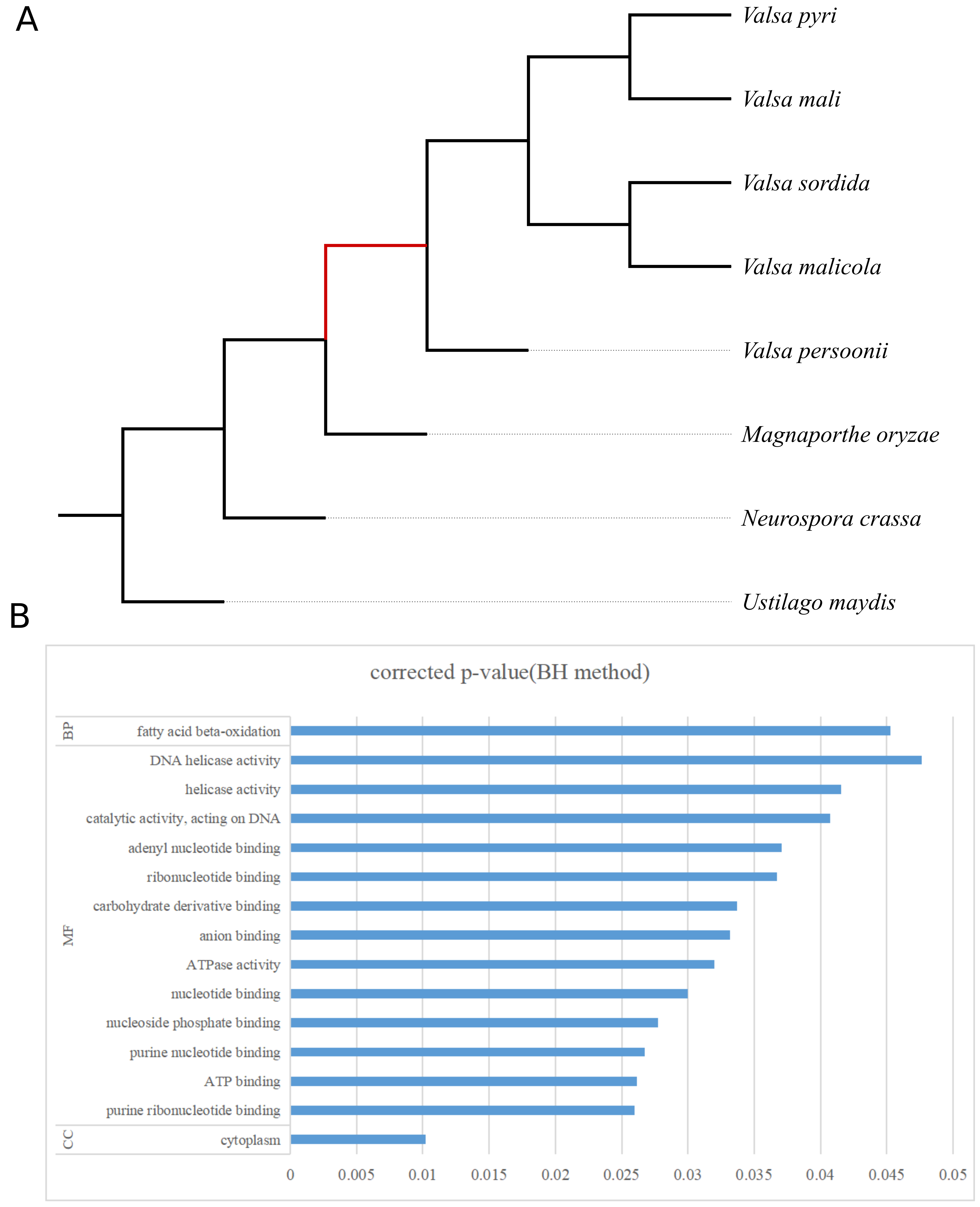

Supplement: jkac312_Supplementary_Data [file jkac312_supplementary_data.zip › Supplementary_Figure_1_G3-2022-403750.png]

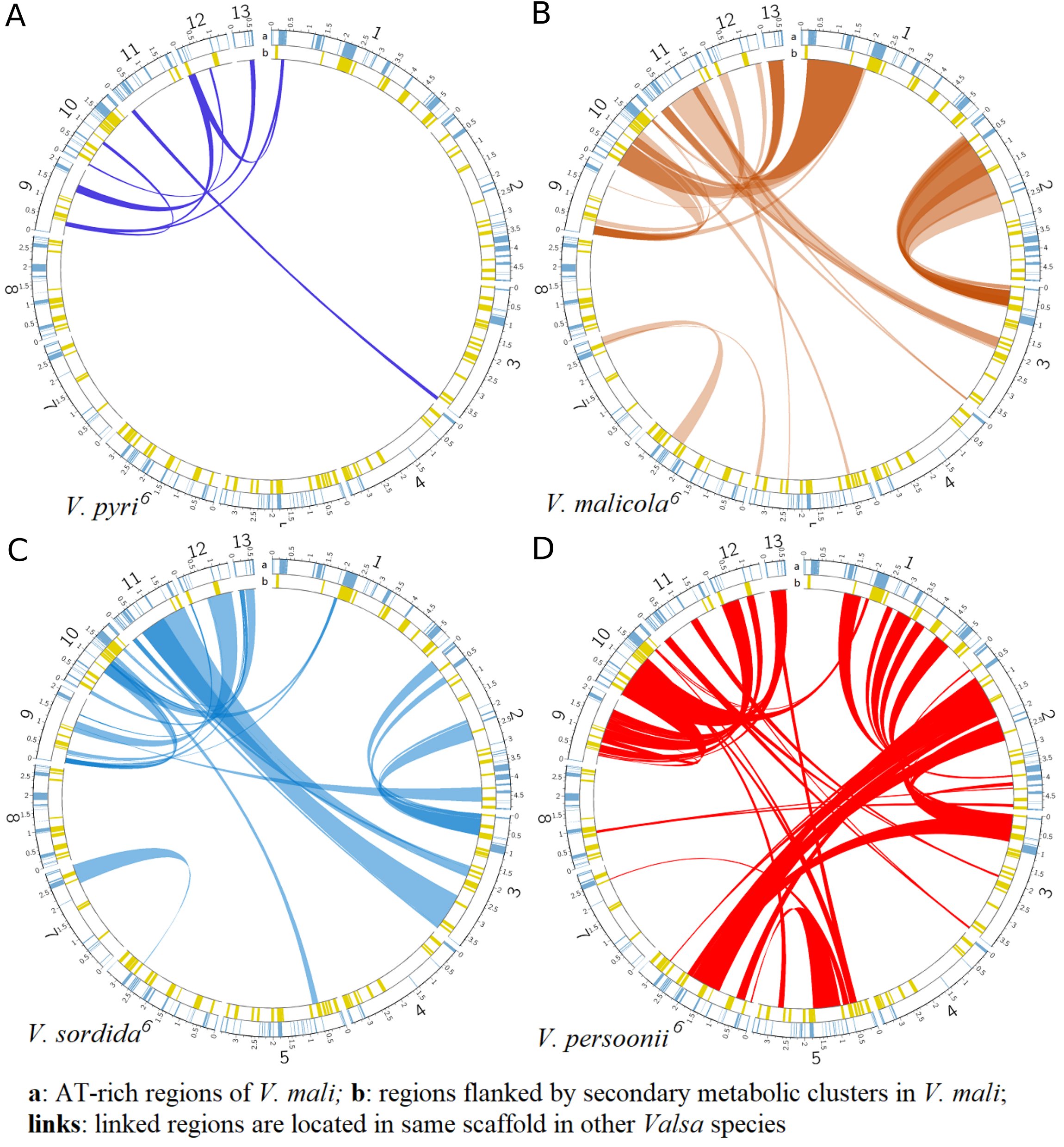

Supplement: jkac312_Supplementary_Data [file jkac312_supplementary_data.zip › Supplementary_Figure_2_G3-2022-403750.png]

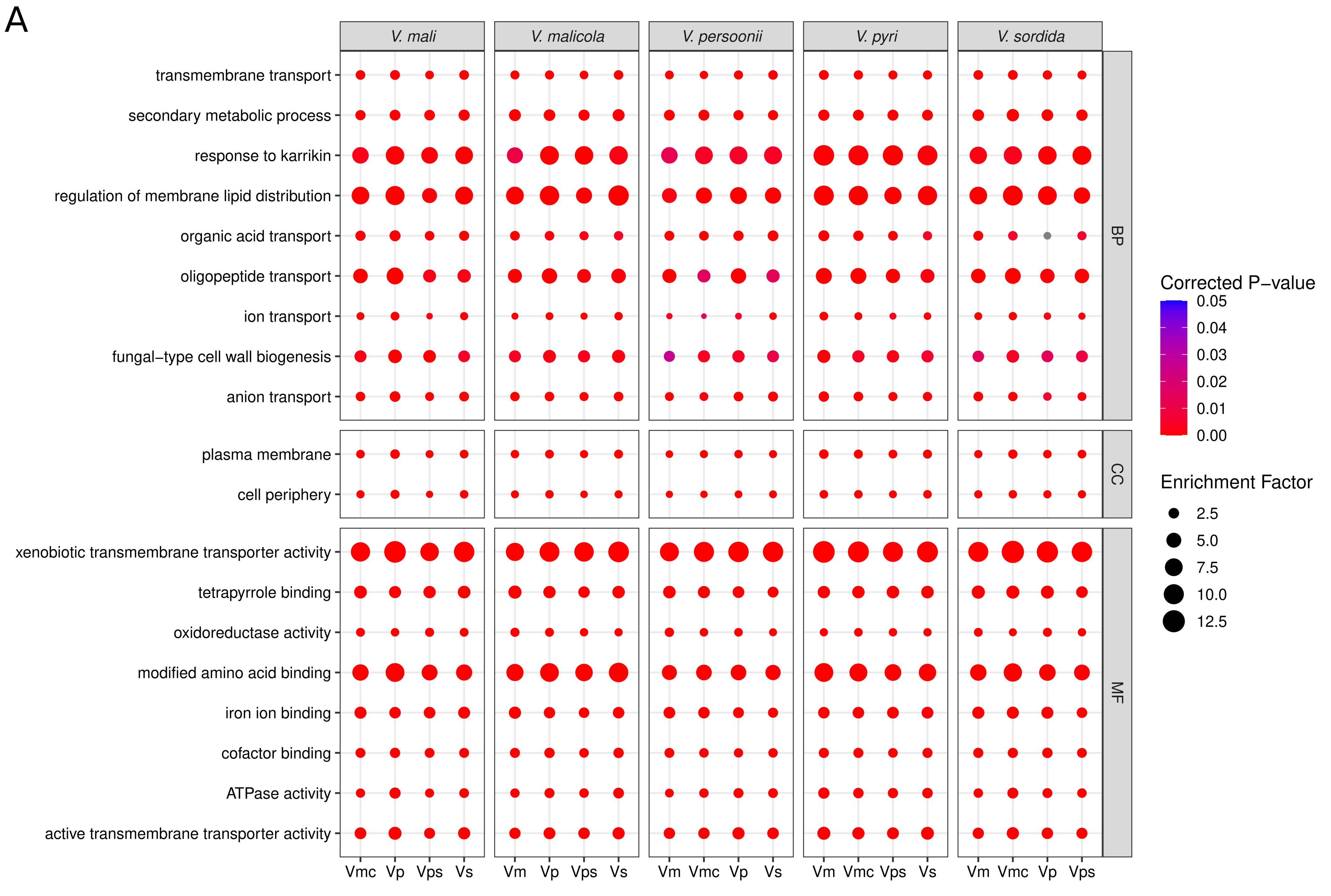

Supplement: jkac312_Supplementary_Data [file jkac312_supplementary_data.zip › Supplementary_Figure_3_G3-2022-403750.png]

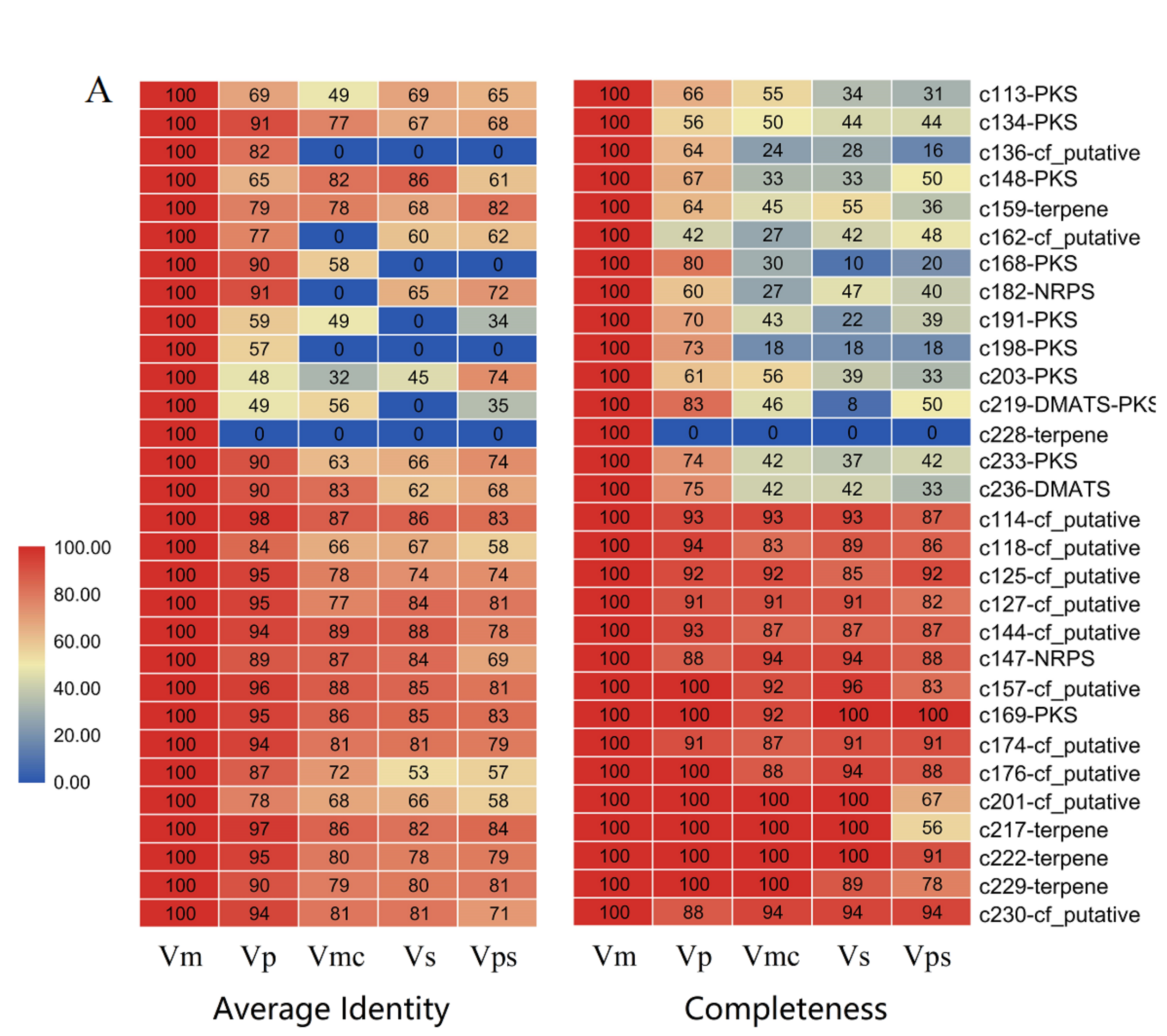

Supplement: jkac312_Supplementary_Data [file jkac312_supplementary_data.zip › Supplementary_Figure_4_G3-2022-403750.png]

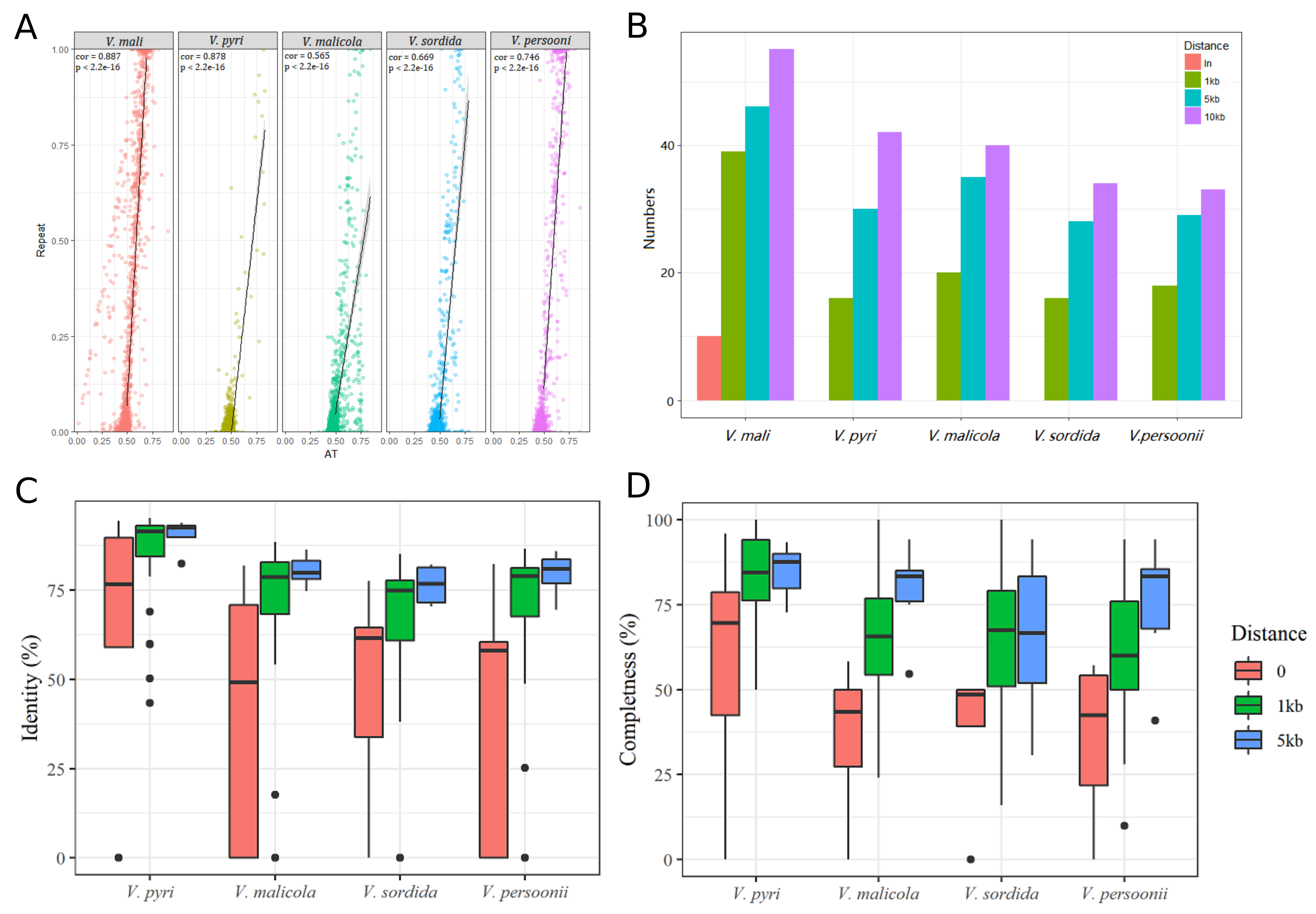

Supplement: jkac312_Supplementary_Data [file jkac312_supplementary_data.zip › Supplementary_Figure_5_G3-2022-403750.png]
